# Genomic analysis of endemic clones of toxigenic and non-toxigenic *Corynebacterium diphtheriae* in Belarus during and after the major epidemic in 1990s

**DOI:** 10.1186/s12864-017-4276-3

**Published:** 2017-11-13

**Authors:** Steffen Grosse-Kock, Valentina Kolodkina, Edward C. Schwalbe, Jochen Blom, Andreas Burkovski, Paul A. Hoskisson, Sylvain Brisse, Darren Smith, Iain C. Sutcliffe, Leonid Titov, Vartul Sangal

**Affiliations:** 1 Faculty of Health and Life Sciences, Northumbria University, Newcastle upon Tyne, UK; 2 Republican Research and Practical Centre for Epidemiology and Microbiology, Minsk, Republic of Belarus; 3 Justus-Liebig-Universität, Gießen, Germany; 4 Friedrich-Alexander-Universität Erlangen-Nürnberg, Erlangen, Germany; 5 Strathclyde Institute of Pharmacy and Biomedical Sciences, University of Strathclyde, Glasgow, UK; 6 Institut Pasteur, Biodiversity and Epidemiology of Bacterial Pathogens, Paris, France

**Keywords:** *Corynebacterium diphtheriae*, Toxigenic, Non-toxigenic, Epidemic, Endemic, Vaccine, Virulence, Diphtheria, Sore throat

## Abstract

**Background:**

Diphtheria remains a major public health concern with multiple recent outbreaks around the world. Moreover, invasive non-toxigenic strains have emerged globally causing severe infections. A diphtheria epidemic in the former Soviet Union in the 1990s resulted in ~5000 deaths. In this study, we analysed the genome sequences of a collection of 93 *C. diphtheriae* strains collected during and after this outbreak (1996 – 2014) in a former Soviet State, Belarus to understand the evolutionary dynamics and virulence capacities of these strains.

**Results:**

*C. diphtheriae* strains from Belarus belong to ten sequence types (STs). Two major clones, non-toxigenic ST5 and toxigenic ST8, encompassed 76% of the isolates that are associated with sore throat and diphtheria in patients, respectively. Core genomic diversity is limited within outbreak-associated ST8 with relatively higher mutation rates (8.9 × 10^−7^ substitutions per strain per year) than ST5 (5.6 × 10^−7^ substitutions per strain per year) where most of the diversity was introduced by recombination. A variation in the virulence gene repertoire including the presence of *tox* gene is likely responsible for pathogenic differences between different strains. However, strains with similar virulence potential can cause disease in some individuals and remain asymptomatic in others. Eight synonymous single nucleotide polymorphisms were observed between the *tox* genes of the vaccine strain PW8 and other toxigenic strains of ST8, ST25, ST28, ST41 and non-toxigenic *tox* gene-bearing (NTTB) ST40 strains. A single nucleotide deletion at position 52 in the *tox* gene resulted in the frameshift in ST40 isolates, converting them into NTTB strains.

**Conclusions:**

Non-toxigenic *C. diphtheriae* ST5 and toxigenic ST8 strains have been endemic in Belarus both during and after the epidemic in 1990s. A high vaccine coverage has effectively controlled diphtheria in Belarus; however, non-toxigenic strains continue to circulate in the population. Recombination is an important evolutionary force in shaping the genomic diversity in *C. diphtheriae*. However, the relative role of recombination and mutations in diversification varies between different clones.

**Electronic supplementary material:**

The online version of this article (10.1186/s12864-017-4276-3) contains supplementary material, which is available to authorized users.

## Background

Diphtheria is a toxin-mediated disease caused by toxigenic strains of *Corynebacterium diphtheriae* which is characterised by the presence of an inflammatory pseudomembrane in the upper respiratory tract, resulting in breathing difficulties with fatal outcomes [1]. Historically, *C. diphtheriae* isolates have been typed phenotypically into four biovars (belfanti, gravis, intermedius and mitis) although genetic approaches have questioned the basis of biovar separation [2]. Diphtheria toxin, which is the most prominent virulence factor of *C. diphtheriae*, inhibits protein synthesis by catalysing NAD^+^-dependent ADP-ribosylation of elongation factor 2, thus inducing apoptosis, resulting in the cell death [3]. The *tox* gene is regulated by a metalloregulatory transcriptional regulator DtxR which induces the toxin production under low iron conditions [4]. The cell death caused by the toxin likely makes the host iron sources available to the pathogen [1]. The toxoid vaccine induces a strong IgG antibody response that neutralises the diphtheria toxin [5] and has approximately 97% efficacy [6]. However, diphtheria remains endemic to many countries [7] and multiple diphtheria outbreaks have been reported across the globe [8–11].

Non-toxigenic *C. diphtheriae* strains are also causing significant invasive infections such as endocarditis, septic arthritis and osteomyelitis [12–14]. These strains lack the *tox* gene, which is present on lysogenising corynephages in toxigenic strains [15]. In addition, non-toxigenic *tox* gene-bearing strains (NTTB) of *C. diphtheriae* are also circulating in the population [16]. The *tox* gene is a pseudogene in NTTB strains due to frameshift mutations but these strains may be able to genetically revert to active toxin production [16].

The major post-vaccine epidemic in the former Soviet Union in the 1990s caused >157,000 cases with approximately 5000 deaths [17]. Belarus, a former Soviet state, reported a significant shift in the distribution of *C. diphtheriae* ribotypes following the epidemic period (between 2000 and 2001) with an increase in the number of infections caused by non-toxigenic strains [18]. This potentially suggests a change in the evolutionary dynamics of *C. diphtheriae* strains. Therefore, to gain a deeper understanding of the population genetics, evolutionary dynamics and virulence capacities, we have sequenced the genomes of a collection of 93 representative toxigenic and non-toxigenic *C. diphtheriae* strains from Belarus isolated between 1996 and 2014 (Additional file 1: Table S1).

## Results

### Major endemic clones of toxigenic and non-toxigenic *C. diphtheriae* in Belarus

A total of 4382 *C. diphtheriae* isolates were collected in Belarus from 1996 to 2014 (Additional file 2: Table S2). Toxigenic strains accounted for approximately 47% of the total isolates in 1996, which fell to zero in 2011. Only non-toxigenic strains have been isolated since 2011 in Belarus. Two to nine isolates from each year were selected for genomic analyses with a proportional representation of toxigenic and non-toxigenic strains (Additional file 2: Table S2). In total, 93 *C. diphtheriae* isolates were selected including one isolate from 1979. These were isolated from all six provinces of Belarus (Brest, Gomel, Grodno, Minsk, Mogilev and Vitebsk) from asymptomatic carriers (*n* = 22) and patients that presented with diphtheria (*n* = 26) or sore throat (*n* = 45). As a part of the clinical diagnosis, these strains were assigned to biovars belfanti, gravis or mitis. The genomes of these strains were sequenced on an Illumina MiSeq instrument and the size of assemblies varied between 2.3-2.6 Mb. Further information on *C. diphtheriae* isolates and genomes assemblies is provided in Additional file 1 (Table S1).

**Fig. 1:**
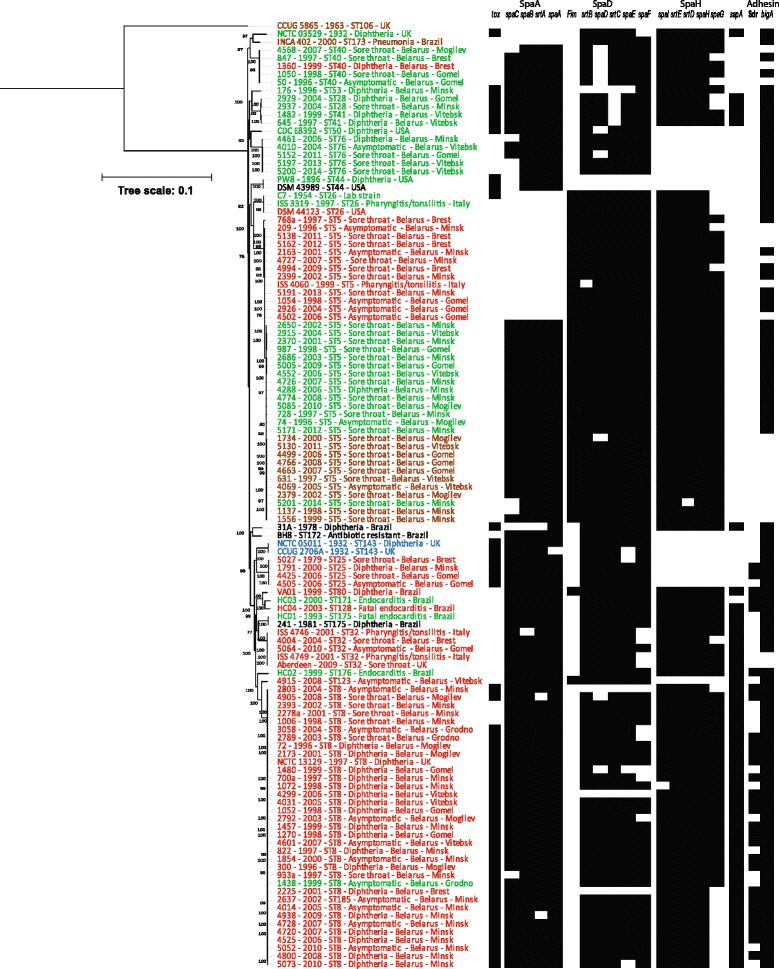
A maximum-likelihood tree derived from concatenated nucleotide sequenced alignment of the core genome. The scale bar represents nucleotide substitutions per nucleotide site. The strain designations of isolates of biovar belfanti, gravis, intermedius and mitis are presented in brown, red, blue and green colour, respectively. The presence of virulence genes is mapped on the tree in black whereas a white box shows the absence of genes

For comparative analyses, genome sequences of two reference strains CCUG 2706A, a strain of rare biovar intermedius, and CCUG 5865, a distinct sequence type (ST)-106 isolate of biovar belfanti, and 22 previously published *C. diphtheriae* strains were also included (Additional file 1: Table S1). The core genome was calculated using EDGAR [19] that is consisted of 1267 genes. A maximum-likelihood (ML) tree from nucleotide sequence alignment of the core genome separated two lineages, one including 116 strains of all four biovars and the second lineage with a single biovar belfanti isolate, CCUG 5865 (Fig. 1). These results support the conclusion of a multilocus sequence typing (MLST) study showing two lineages within *C. diphtheriae* [20].

The majority (76%) of *C. diphtheriae* isolates from Belarus formed two groups within lineage 1, ST5 (37 isolates, 39.8%) and ST8 (34 isolates, 36.6%; Fig. 1). ST5 is a non-toxigenic clone while most isolates in ST8 are toxigenic. Toxigenic ST8 isolates were responsible for the epidemic in the former Soviet Union in the 1990s [17, 20] and this study reveals that these strains were also circulating after the epidemic period (Additional file 1: Table S1).

Two non-toxigenic isolates were ST32 that are known to cause severe pharyngitis and tonsillitis among patients in Europe [21, 22]. Other minor groups include toxigenic ST25 (4 isolates) and ST28-ST41 (4 isolates), nontoxigenic ST76 (5 isolates) and NTTB ST40 (5 isolates). Two strains, one toxigenic ST53 and one nontoxigenic ST123, are singletons.

### Spatio-temporal distribution of *C. diphtheriae* clones

**Fig. 2:**
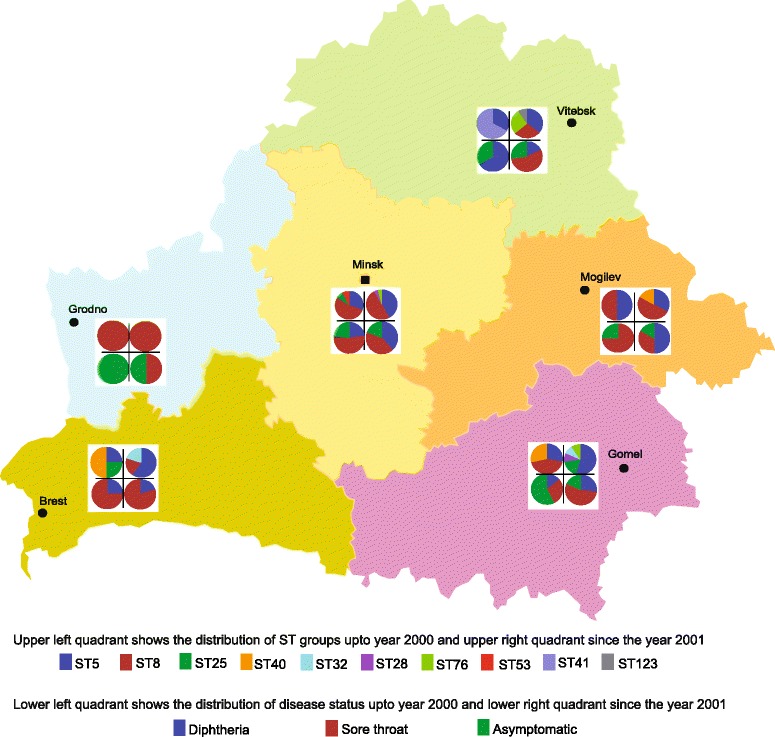
A distribution of genotypes (STs) and disease states (asymptomatic, diphtheria and sore throat) among *C. diphtheriae* isolates in difference provinces of Belarus

To investigate the reported shift in the major genotypes between 2000 and 2001 in Belarus [18], we analysed the temporal distribution of *C. diphtheriae* strains in Belarus predating 2001 (epidemic period) and since January 2001 (post-epidemic period; Fig. 2). ST5 and ST8 strains have been prevalent in most of the Belarussian provinces in both the epidemic and post-epidemic periods and we did not observe any shift in the distribution of strains in these STs (χ^2^ test, *p* > 0.05; Additional file 2: Table S3) to correlate with the replacement of biovar gravis by mitis and change in the distribution of ribotypes [18]. Ribotyping is error prone as the resolution and reproducibility are dependent on multiple factors including the restriction enzymes and stringency of the hybridisation conditions [23]. We have previously shown that biovar designations are not necessarily reliable and are not supported by genomic diversity [24] which is strengthened by the fact that ST5 includes strains assigned to biovars belfanti, gravis and mitis (Fig. 1). Although biovars belfanti and mitis isolates formed a subgroup (ST5-B; Additional file 3: Figure S1) within ST5, the dataset does not indicate any replacement of gravis isolates by mitis as the strains belonging to these biovars were isolated both during and after the epidemic period. These findings further suggest that genetic approaches should be adopted over biotyping for studying *C. diphtheriae* epidemiology.

At the provincial level, all three isolates were ST8 in Grodno province in both the periods (Fig. 2). In some provinces, certain clones were observed either in the epidemic (ST8 in Gomel, ST25 in Brest and Minsk, ST40 in Brest and Gomel, and ST41 in Vitebsk) or in the post-epidemic period only (ST8 in Brest and Vitebsk, ST25 in Gomel, and ST40 in Mogilov; Fig. 2). Although ST25 and ST40 strains are rare, they seem to be maintaining a reservoir as they appeared in some provinces in the epidemic period and in other provinces in the post-epidemic period. Some clones were only observed in the post-epidemic period; for example, ST28 in Gomel and Minsk, and ST32 in Brest and Gomel. It is possible that these strains were introduced in Belarus after 2001. Alternatively, these rare strains may have also been circulating prior to year 2000 but a larger sample size from the epidemic period needs be analysed to detect them.

### Asymptomatic carriage and disease status

Of the 93 isolates from Belarus, 22 (23.7%) were isolated from asymptomatic carriers, 45 (48.4%) from patients with a sore throat and only 26 (28.0%) were from diphtheria patients (Additional file 1: Table S1; Fig. 2). The diseases status is clearly associated with different *C. diphtheriae* clones (χ^2^ test, *p* < 0.001; Additional file 2: Table S4). The majority of the ST5 strains (29/37 isolates; 78.4%) caused sore throat and 7 strains (18.9%) were associated with asymptomatic carriage. Although all ST5 isolates are non-toxigenic, one isolate caused diphtheria-like symptoms in a patient. 29.4% (10/34 isolates) ST8 isolates were carriage-associated, 52.9% (18/34) caused diphtheria and 17.7% (6/34) caused sore throat. ST8 isolates are toxigenic except for three that are non-toxigenic isolated from the patients with sore throat.

Similarly, isolates of minor *C. diphtheriae* clones, toxigenic ST25 and non-toxigenic ST40 and ST76, were isolated from healthy carriers as well as from patients with sore throat, diphtheria or diphtheria-like symptoms (Figs. 1 & 2). Non-toxigenic ST32 isolates were either asymptomatic or caused sore throat whereas isolates of the ST28-ST41 group caused sore throat or diphtheria in patients. Interestingly, strains of the same ST have the ability to asymptomatically inhabit the human respiratory tract or to cause sore throat and diphtheria or diphtheria-like symptoms (non-toxigenic strains), regardless of their toxigenicity.

### Clonal expansion of major *C. diphtheriae* clones

To understand the mechanism of clonal expansion in *C. diphtheriae*, we focused on the major clones, non-toxigenic ST5 and toxigenic ST8. Overall, 94,033 single nucleotide polymorphisms (SNPs) were observed within the core genomic alignment (1,226,854 bp) of 117 isolates. 3577 SNPs were present within ST5 whereas only 426 SNPs were present among ST8 isolates. The concatenated core genomic alignment was analysed by Gubbins [25] that indicated higher diversity being introduced by recombination than point mutations at the internal branch level which is shared by all the isolates within each group (Additional file 3: Figure S2). Gubbins identifies the regions introduced by recombination in the whole genomic alignments and calculates relative frequencies of recombination and mutations in clonal diversification. *C. diphtheriae* genomes analysed in this study are draft assemblies with some gaps. We did not attempt to predict genome-wide recombination rates and only focused on identification of regions introduced by recombination in the core genome of *C. diphtheriae*.

The regions predicted to be acquired through recombination were removed from the core genomic alignment, resulting in an alignment of 806,921 bp for ST5 (414 SNPs) and 861,883 bp for ST8 isolates (263 SNPs). Therefore, ST5 isolates acquired more diversity among the core genes through recombination than ST8.The level of temporal signal slightly varied between ST5 and ST8 after stripping the imported regions (Additional file 3: Figure S3). The correlation between the root-to-tip distances and strain isolation dates was relatively stronger within ST8 (R^2^ = 0.501) than ST5 (R^2^ = 0.310) with core genomic clock-rates of 8.9 × 10^−7^ (95% highest posterior density interval 5.6 × 10^−7^ – 1.2 × 10^−6^) and 5.6 × 10^−7^ (95% highest posterior density interval 3.7 – 7.7 × 10^−7^) substitutions per strain per year, respectively. Therefore, point mutations are slightly more frequent in ST8 than ST5.

### Virulence potential of *C. diphtheriae* clones

The genome sequences are quite conserved within each clone which is reflected in the CDS BLAST maps of ST5 and ST8 (Additional file 3: Figure S4A-B). 1807 genes (81-87% genes in individual isolates) were shared by all ST5 isolates and 1770 genes (78-86% genes) were common within ST8. A ML-tree from the accessory genome retrieved similar groupings as the core genome but indicated minor variations in the gene content that may result in functional variations between individuals of a clone (Additional file 3: Figure S5). The strains assigned to subgroup ST5-A and ST5-C possessed only two pilus gene clusters, SpaD and SpaH, whereas an additional SpaA cluster was present among the strains in ST5-B (Fig. 1; Additional file 3: Figure S1). Pilus gene clusters are borne by genomic islands [26] and ST5-B may have horizontally acquired the SpaA gene cluster from other *C. diphtheriae* strains. In addition, some genes in the pilus gene clusters have lost their function due to frameshift mutations; for example, the *spaC* gene in the SpaA gene cluster of strains 5201 and 1137. A gene encoding BigA-like adhesin which is known to mediate adhesion to epithelial cells [27] was possessed by some ST5 isolates, both in subgroups ST5-A and ST5-B (Fig. 1). Therefore, recombination and gain or loss of gene functions are introducing functional variations among isolates within a single clone [21].

Most of the ST8 isolates are equipped with all three Spa gene clusters, except for three strains that lacked the SpaD cluster. Similar to ST5, some genes in different *spa* clusters were pseudogenes. All ST8 isolates possessed gene encoding BigA-like adhesin (DIP2014) and an additional gene, *sapA* (DIP2066), encoding a surface-anchored pilus protein which is absent in ST5 isolates. In addition, most of the ST8 isolates carried another gene (DIP2093) encoding an adhesin of the Sdr family (Fig. 1). Similarly, the numbers and organisation of *spa* clusters and the presence/absence of *sapA* and adhesin genes varied both within and between other clones (Fig. 1). As reported in the previous sections, individual isolates of the same clone can cause different pathologies in different individuals. It is possible that gain or loss of the gene functions in pilus gene clusters or other virulence genes is partially contributing to the degree of disease; however, such a correlation is not obvious as some isolates from asymptomatic carriers have the *tox* gene along with all the above-mentioned virulence genes.

**Fig. 3:**
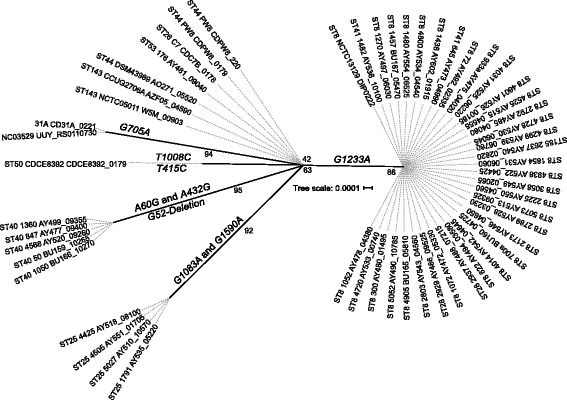
A ML tree from the nucleotide sequence alignment of the *tox* gene. The scale bar represents the number of nucleotide substitutions per site. SNPs separating the clones from the vaccine strain PW8 are mapped on the branches

The key virulence factor in *C. diphtheriae* is the *tox* gene which is present among ST8 (except for three isolates), ST25 and ST28-ST41 isolates in Belarus. Interestingly, ST40 isolates were NTTB strains where a deletion of a nucleotide at position 52 in the *tox* gene resulted in the frameshift. A total of eight SNPs were observed between the *tox* gene of vaccine strain PW8 and other toxigenic strains in the dataset but all of them are synonymous (Fig. 3), suggesting that the impact of the vaccine will be similar on all toxigenic isolates.

## Discussion

*C. diphtheriae* is genetically diverse with >11 distinct groups identified by the analysis of MLST data [2]. Most of the isolates from Belarus belong to two major clones, ST5 and ST8, with the remaining isolates distributed to eight other STs (Fig. 1). ST5 and ST8 strains from Belarus vary in their virulence gene repertoire and differ in their ability to cause disease (Fig. 1). ST5 isolates lack the *tox* gene, *sapA* and Sdr-like adhesin with additional absence of the SpaA gene cluster among subgroup ST5-A and ST5-C isolates. SpaA pili are responsible for adhesion to pharyngeal epithelial cells and SpaD and SpaH pili interact with laryngeal and lung epithelial cells [28, 29]. Sdr-like adhesin (DIP2093) also helps the pathogen in interacting with host cells and biofilm formation [30, 31]. Therefore, ST8 isolates may have greater abilities to adhere and invade host cells in comparison to ST5 isolates. However, regardless of the virulence potential, *C. diphtheriae* strains can cause disease in some individuals, while others remain asymptomatic. These asymptomatic carriers may serve as reservoir for dissemination of the pathogen to the community [32].

The toxin is responsible for the cell death which is produced under low iron conditions [3, 4]. Iron is essential for growth of all organisms and pathogenic bacteria often rely on the host for iron supply [33]. Most of the genes involved in iron uptake and transport including Irp6A-C (DIP0108-DIP0110), DIP0582-0586, HmuT-V (DIP0626-0628) and DIP1059-1062 are conserved in *C. diphtheriae* with minor exceptions. However, ChtC-CirA (DIP0522-DIP0523), ChtAB (DIP1519-DIP1520) and HtaA-C (DIP0624, DIP0625 and DIP0629) that are involved in uptake of hemoglobin-haptoglobin complexes [34], are only present in 48-73 strains. Interestingly, the majority of ST8 isolates possess these genes, suggesting that they are better equipped to utilise iron from the host cells than ST5 isolates.

MLST studies analysing *C. diphtheriae* strains from Russia and Poland also revealed the presence of diverse strains in these neighbouring countries during the epidemic period [14, 20]. However, ST8 isolates were apparently more prevalent in Poland in the post-epidemic period [14]. Consistent with the present study, all ST8 isolates from Russia belonged to biovar gravis and were toxigenic and ST5 isolates were non-toxigenic [20]. Invasive ST8 isolates in Poland were also biovar gravis but they were non-toxigenic [14]. A high diphtheria vaccination coverage in Poland probably protected the population from the epidemic in the neighbouring Soviet States in the 1990s [14, 35]. A consistency between *C. diphtheriae* ribotypes and grouping from other typing approaches has been previously reported [14, 20, 36]. Ribotyping information was available for 50 of the 93 strains from Belarus and we looked at their distribution within *C. diphtheriae* clones. In agreement with previous findings, epidemic ST8 clone encompassed ribotypes D1 and D4; however, one isolate each of ribotypes D6 and D7 were also present in this group (Additional file 1: Table S1) [37–39]. All D10 ribotype isolates were confined to ST5, whereas all isolates within ST25 were ribotype D6. Ribotypes of ST40 isolates were unclear except for a single isolate which was identified as D4. ST41 included one isolate each of ribotype D7 and D8 and one D7 isolate fell within ST53. ST76 isolate was identified to be a new (unassigned) ribotype. Therefore, ribotyping is generally concordant with the MLST and genomic groupings, with some exceptions.

The diversity at the clustered regularly interspaced short palindromic repeat (CRISPR) loci has been used to characterise *C. diphtheriae* outbreaks [37–39]. 20 *C. diphtheriae* isolates from Belarus belonging to ribotype D4 were divided into three spoligotypes based on the diversity at both the DRA and DRB CRISPR loci [39]. We have previously highlighted the extensive diversity at CRISPR loci between different *C. diphtheriae* strains based on the direct repeat and spacers sequences extracted from the genome sequences [40]. In this study, 16 combinations of direct-repeats and spacers are observed at the DRA locus among ST8 isolates while this locus is absent in one strain (Additional file 2: Table S5). The DRB locus was more diverse among these isolates with 20 direct-repeat and spacer combinations, resulting in a total of 30 combined profiles among 35 ST8 isolates (Additional file 2: Table S3). These findings are consistent with the previous studies revealing 45 combined spoligotypes among epidemic *C. diphtheriae* isolates in Russia [37, 38]. The DRB locus was absent among ST5 isolates and they were subdivided into 21 CRISPR types based on the diversity at the DRA locus (Additional file 2: Table S5).

Interestingly, the evolutionary dynamics of the non-toxigenic clone ST5 varied from the toxigenic clone ST8, with recombination being more prevalent within ST5, particularly in subgroup ST5-B (Additional file 3: Figure S2). *C. diphtheriae* inhabits the human upper respiratory tract which is also a niche for a variety of other bacteria [41], providing opportunities for recombination. Indeed, recombination frequencies are high among bacteria in the upper respiratory tract [42]. This variation in the relative role of recombination and point mutation in diversification of toxigenic and non-toxigenic strains is interesting and confirms that different lineages of the same species may have different recombination and mutation rates [43]. It is also possible that vaccine-induced immune response may be influencing the evolutionary dynamics by applying a selective pressure to toxigenic ST8 isolates. Vaccination was found to affect the evolution in *Bordetella pertussis* where the molecular clock rate was associated with the vaccination coverage in different countries [44]. It will be interesting if more studies characterising genomic variations in the collection of other toxigenic and non-toxigenic *C. diphtheriae* clones also observe similar differences in the evolutionary dynamics.

A high coverage of diphtheria vaccine in Belarus has significantly reduced the number of diphtheria cases and no new cases have been reported to WHO since 2011 [7] (Additional file 3: Figure S6). However, infections caused by non-toxigenic strains continue to emerge in most Belarusian provinces (Additional file 1: Table S1). IgG antibodies induced by the vaccine neutralise the toxin; however, it is unclear if the vaccine eliminates the organism or not. The toxoid vaccine for botulism reduced free neurotoxin in cows as well as the number of *Clostridium botulinum* spores in the faeces [45]. It is possible that neutralising antibodies eliminate the pathological effects of the toxin that potentially allow for the development of an adaptive immune response to limit growth of the bacteria. Several membrane-associated and secreted proteins have been detected in diphtheria vaccines by highly sensitive mass-spectrometry (Möller and Burkovski, unpublished data). These proteins may stimulate production of antibodies against additional targets on the cell surface. Therefore, the vaccine may be more effective against toxigenic strains but may also target non-toxigenic strains.

## Conclusions

In conclusion, the diphtheria vaccine remains effective against toxigenic strains and has largely controlled diphtheria in Belarus after the major epidemic in the 1990s. This study describes the diversity among *C. diphtheriae* strains that were circulating in the period from 1996 to 2014 and demonstrates variation in the evolutionary dynamics between the two prevalent *C. diphtheriae* clones in Belarus. The variation in the presence of virulence genes and gain or loss of gene function is likely responsible for the differences in virulence characteristics, not only between different clones but also between isolates within a single clone. Regardless of their virulence potential, *C. diphtheriae* strains can asymptomatically colonise some individuals which exacerbates the threat of dissemination to the wider community.

## Methods

### Bacterial strains

The details of 93 *C. diphtheriae* isolates from Belarus are presented in Additional file 1 (Table S1). Two reference strains CCUG 2706A and CCUG 5865 were obtained from the CCUG Culture Collection, Göteborg, Sweden.

### Genome sequencing

All 95 isolates (93 isolates from Belarus and two reference strains) were cultured on Brain-Heart Infusion agar overnight at 37 °C and a single colony was used to inoculate a 5 ml Brain-Heart Infusion broth. DNA was extracted from 2 ml overnight culture incubated at 37 °C for 16 h in a shaking incubator using the UltraClean® Microbial DNA Isolation Kit (MoBio). The genomes were sequenced on an Illumina MiSeq instrument and the reads were assembled using the CLC Genomic Workbench (Qiagen) or SPAdes 3.9.0 [46]. Genome assemblies were submitted to the GenBank for annotation by the NCBI Prokaryotic Genome Annotation Pipeline [47]. The genomes sequences of 22 previously published *C. diphtheriae* strains were obtained from the GenBank (Additional file 1: Table S1).

### Comparative genomic and phylogenetic analyses

A comparative analysis on the complete dataset of 117 genomes was performed using EDGAR [19]. Orthologs of virulence genes including *sapA* (DIP2066), adhesin of Sdr family (DIP2093) and BigA-like adhesin (CDC7B_1983; DIP2014) were searched within the dataset using EDGAR. The CDS BLAST maps were generated using the CGView Comparison Tool [48] for isolates in ST5 and ST8 using concatenated sequence of ISS 4060 and NCTC 13129 as the reference, respectively. MLST profiles were extracted from the genome sequences using MLST 1.8 [49]. Single locus variants to known sequence types were treated as the same clone (ST).

The nucleotide sequences of the concatenated core genome were aligned using MUSCLE [50] and poorly aligned regions were removed by GBLOCKS [51]. A ML tree from the core genomic alignment was generated using GTR + I + G4 model according to Bayesian Information Criterion (BIC) with 100,000 SH-like approximate likelihood ratio tests (SH-aLRT) and 100,000 ultrafast bootstrap iterations using IQ-Tree [52]. A ML tree from the binary data of the presence or absence of genes in the accessory genome was generated using GTR2 + FO + ASC + R5 model with 1000 SH-aLRT and 1000 ultrafast bootstrap iterations. Both the trees were re-rooted using the strain CCUG 5865. ML tree were generated separately from the core genome of ST5 isolates using TIM + I model and from the nucleotide sequence alignment of *tox* gene using the HKY model, each with 100,000 SH-aLRT and 100,000 ultrafast bootstrap iterations. All phylogenetic trees were visualized using iTOL [53].

### Spatio-temporal association of *C. diphtheriae* clones

χ^2^ tests were performed to analyse differential distribution of *C. diphtheriae* strains in ST5 and ST8 clones in epidemic and post epidemic periods and their association with the disease status using the package SPSS v24 (IBM).

### Evolutionary analyses

The regions introduced by recombination in the core genome were identified using Gubbins [25] and were masked from the alignment using the script maskrc-svg.py (provided by Kwong, J. and Seemann, T; https://github.com/kwongj/maskrc-svg). ML trees were constructed using IQ-Tree [52] with the best-fit substitution models and 100,000 SH-aLRT and 100,000 ultrafast bootstrap iterations from the core genomic alignment of Belarussian ST5 and ST8 isolated after stripping the masked regions. These phylogenetic trees were analysed by tempEST v 1.5 with the sampling dates and best-fit root criteria to detect temporal signal [54]. The clock-rates were calculated from these alignments using BEAST [55]. The HKY substitution model was used with Coalescent Bayesian Skyline and 10,000,000 MCMC chain length and 10,000 burn-in iterations. The trace file was analysed using Tracer v1.6 [56].

## Additional files

Additional file 1: Table S1. Details of *C. diphtheriae* strains analysed in this study. (XLSX 21 kb)

Additional file 2: Table S2. Number and toxigenicity of isolates collected between 1996 and 2014 in Belarus and those selected for genomic analyses in this study; **Table S3.** Distribution of isolates from epidemic (≤ year 2000) and postepidemic period (≥ year 2001) in major groups. All minor groups are pooled together for statistical analysis; **Table S4.** Distribution of isolates from asymptomatic carriage, diphtheria and sore throat patients in major groups. All minor groups are pooled together for statistical analysis; **Table S5.** Allelic variation in the direct repeat and spacer sequences among the CRISPR loci of ST8 and ST5 isolates. (PDF 396 kb)

Additional file 3: Figure S1. A ML tree from core genomic alignment of ST5 strains; **Figure S2.** Gubbins analysis of recombination in the core genome of *C. diphtheriae*. Predicted recombination events on internal branches are shown in red and those occurred at terminal branches are shown in blue; **Figure S3.** A plot of root-to-tip divergence (Y-axis) against the sampling dates (X-axis); A. within ST5 and B. within ST8; **Figure S4. A.** CDS BLAST map of ST5 using strain ISS 4060 as the reference. **B.** CDS BLAST map of ST8 using strain NCTC 13129 as the reference; **Figure S5.** A ML tree from the binary data of the presence or absence of genes in the accessory genome. The scale bar with a distance of 0.1 represents the difference of 341.7 genes; **Figure S6.** A plot showing the average global vaccine coverage, reported vaccination in Belarus and the reported number of diphtheria cases between 1992 and 2015. (PDF 5948 kb)

## Abbreviations

BLAST: Basic local alignment search tool
CDS: Coding DNA Sequence
IgG: Immunoglobulin G
MCMC: Markov chain Monte Carlo
ML tree: Maximum-Likelihood tree
MLST: Multilocus sequence typing
NTTB: Non-toxigenic *tox* gene-bearing strains
SH-aLRT: SH-like approximate likelihood ratio test
ST: Sequence type
