# Supplementary material for: Genomic analysis of endemic clones of toxigenic and non-toxigenic Corynebacterium diphtheriae in Belarus during and after the major epidemic in 1990s
Source: BMC Genomics. 2017 Nov 13;18:873. doi: 10.1186/s12864-017-4276-3 (PMC5683216; doi:10.1186/s12864-017-4276-3)
Supplement: Supplementary file 2 — Number and toxigenicity of isolates collected between 1996 and 2014 in Belarus and those selected for genomic analyses in this study; Table S3. Distribution of isolates from epidemic (≤ year 2000) and postepidemic period (≥ year 2001) in major groups. All minor groups are pooled together for statistical analysis; Table S4. Distribution of isolates from asymptomatic carriage, diphtheria and sore throat patients in major groups. All minor groups are pooled together for statistical analysis; Table S5. Allelic variation in the direct repeat and spacer sequences among the CRISPR loci of ST8 and ST5 isolates. (PDF 396 kb) [file 12864_2017_4276_MOESM2_ESM.pdf]

Additional File 2: Table S2. Number and toxigenicity of isolates collected between 1996 – 2014 in Belarus and those selected for genomic analyses in this study

	Total isolates			Selected for this study		
**Year**	**No. of isolates**	**tox+ (%)**	**tox- (%)**	**No. of isolates**	**tox+ (%)**	**tox- (%)**
1996	486	47.12	52.88	6	50.00	50.00
1997	410	36.34	63.66	8	50.00	50.00
1998	362	19.61	80.39	8	37.50	62.50
1999	339	30.68	69.32	6	66.67	33.33
2000	403	29.78	70.22	3	33.33	66.67
2001	408	13.48	86.52	5	40.00	60.00
2002	310	8.06	91.94	5	20.00	80.00
2003	213	7.51	92.49	3	66.67	33.33
2004	290	19.31	80.69	8	37.50	62.50
2005	323	6.19	93.81	3	66.67	33.33
2006	201	3.48	96.52	9	55.56	44.44
2007	204	2.94	97.06	7	42.86	57.14
2008	158	5.70	94.30	5	40.00	60.00
2009	98	3.06	96.94	3	33.33	66.67
2010	77	5.19	94.81	4	50.00	50.00
2011	54	0.00	100.00	3	0.00	100.00
2012	21	0.00	100.00	2	0.00	100.00
2013	10	0.00	100.00	2	0.00	100.00
2014	15	0.00	100.00	2	0.00	100.00

Additional File 2: Table S3. Distribution of isolates from epidemic (≤ year 2000) and post-epidemic period (≥ year 2001) in major groups. All minor groups are pooled together for statistical analysis.

Sampling period	Groups			Total
	ST5	ST8	Other	
Epidemic period	10	13	9	32
Post-epidemic period	27	21	13	61
Total	37	34	22	93

Chi square statistics on above distribution:

	Value	df	Asymptotic Significance (2-sided)	Monte Carlo Sig. (2-sided)		
				Significance	99% Confidence Interval	
					Lower Bound	Upper Bound
Pearson Chi-Square	1.526[a]	2	0.466	.533[b]	0.52	0.546
Likelihood Ratio	1.548	2	0.461	.533[b]	0.52	0.546
Fisher's Exact Test	1.579			.533[b]	0.52	0.546
N of Valid Cases	93					

a. 0 cells (0.0%) have expected count less than 5. The minimum expected count is 5.20.

b. b. Based on 10000 sampled tables with starting seed 1314643744.

Additional File 2: Table S4. Distribution of isolates from asymptomatic carriage, diphtheria and sore throat patients in major groups. All minor groups are pooled together for statistical analysis.

Groups	Clinical information			Total
	Asymptomatic	Diphtheria	sore throat	
ST5	7	1	29	37
ST8	10	18	6	34
Other	5	7	10	22
Total	22	26	45	93

Chi square statistics on above distribution:

	Value	df	Asymptotic Significance (2-sided)	Monte Carlo Sig. (2-sided)		
				Significance	99% Confidence Interval	
					Lower Bound	Upper Bound
Pearson Chi-Square	30.542[a]	4	0	.000[b]	0.000	0.000
Likelihood Ratio	35.572	4	0	.000[b]	0.000	0.000
Fisher's Exact Test	33.655			.000[b]	0.000	0.000
N of Valid Cases	93					

a. 0 cells (0.0%) have expected count less than 5. The minimum expected count is 5.20.

b. Based on 10000 sampled tables with starting seed 299883525.

Additional File 2: Table S5. Allelic variation in the direct repeat and spacer sequences among the CRISPR loci of ST8 and ST5 isolates

Strain	Year	Biovar	Clinical information	Country	DRA type	DRA locus	DRB type	DRB locus
1480	1999	Gravis	Diphtheria	Gomel	CT2-1	D1-S111-S108	CT1E-12	D4-S97-S7-S65-S82-S8-S17-S58-S106-S54-S19-S62-S86-S42-S98-S46-S70-S64-S66-S91
4905	2008	Gravis	Sore throat	Mogilev	CT2-2	D1-S112-S27-S80-S103-S41-S111-S107	CT1E-1	D4-S97-S17-S58-S106-S54-S28-S2-S85-S71-S28-S2-S86-S85-S71-S61-S42-S98-S46-S70-S64-S5-S8
2637	2002	Gravis	Asymptomatic	Minsk	CT2-3	D1-S13-S27-S80-S103-S41-S111-S107	CT1E-20	D5-S95-S53-S57-S32-S39-S83-S48-S72-S14-S115-S78-S20-S74-S90-S94-S26-S18-S81-S24-S29-S43-S38
4601	2007	Gravis	Asymptomatic	Vitebsk	CT2-4	D1-S27-S80-S103-S41-S111-S107	CT1E-4	D4-S97-S65-S5-S8-S17-S58-S106-S54-S19-S28-S42-S98-S46-S70-S64-S66-S91-S7
300	1996	Gravis	Diphtheria	Mogilev	CT2-4	D1-S27-S80-S103-S41-S111-S107	CT1E-6	D4-S97-S7-S65-S82-S51-S36-S5-S8-S17-S58-S106-S86-S54-S19-S28-S2-S71-S28-S2-S85-S71-S61-S42-S98-S46-S70-S64-S66-S91
1270	1998	Gravis	Diphtheria	Gomel	CT2-4	D1-S27-S80-S103-S41-S111-S107	CT1E-6	D4-S97-S7-S65-S82-S51-S36-S5-S8-S17-S58-S106-S86-S54-S19-S28-S2-S71-S28-S2-S85-S71-S61-S42-S98-S46-S70-S64-S66-S91
1457	1999	Gravis	Diphtheria	Minsk	CT2-4	D1-S27-S80-S103-S41-S111-S107	CT1E-6	D4-S97-S7-S65-S82-S51-S36-S5-S8-S17-S58-S106-S86-S54-S19-S28-S2-S71-S28-S2-S85-S71-S61-S42-S98-S46-S70-S64-S66-S91
1854	2000	Gravis	Asymptomatic	Minsk	CT2-4	D1-S27-S80-S103-S41-S111-S107	CT1E-6	D4-S97-S7-S65-S82-S51-S36-S5-S8-S17-S58-S106-S86-S54-S19-S28-S2-S71-S28-S2-S85-S71-S61-S42-S98-S46-S70-S64-S66-S91
4031	2005	Gravis	Diphtheria	Vitebsk	CT2-4	D1-S27-S80-S103-S41-S111-S107	CT1E-7	D4-S97-S7-S65-S82-S51-S36-S5-S8-S17-S58-S106-S86-S54-S19-S28-S2-S85-S71-S61-S42-S98-S46-S70-S64-S66-S91
822	1997	Gravis	Diphtheria	Minsk	CT2-4	D1-S27-S80-S103-S41-S111-S107	CT1E-9	D4-S97-S7-S65-S82-S51-S36-S5-S8-S17-S58-S86-S42-S98-S46-S70-S64-S66-S91
2173	2001	Gravis	Diphtheria	Mogilev	CT2-4	D1-S27-S80-S103-S41-S111-S107	CT1E-10	D4-S97-S7-S65-S82-S8-S17-S58-S106-S54-S19-S28-S86-S2-S85-S71-S28-S2-S85-S71-S61-S42-S98-S46-S70-S64-S66-S91
3058	2004	Gravis	Asymptomatic	Grodno	CT2-4	D1-S27-S80-S103-S41-S111-S107	CT1E-11	D4-S97-S7-S65-S82-S8-S17-S58-S106-S54-S19-S28-S86-S2-S85-S71-S61-S42-S98-S46-S70-S64-S66-S91
72	1996	Gravis	Diphtheria	Mogilev	CT2-4	D1-S27-S80-S103-S41-S111-S107	CT1E-14	D5-S26-S48-S72-S14-S115-S78-S74-S90-S94-S18-S81-S24-S29-S57-S32-S39-S83
1052	1998	Gravis	Diphtheria	Gomel	CT2-4	D1-S27-S80-S103-S41-S111-S107	CT1E-20	D5-S95-S53-S57-S32-S39-S83-S48-S72-S14-S115-S78-S20-S74-S90-S94-S26-S18-S81-S24-S29-S43-S38
2792	2003	Gravis	Asymptomatic	Mogilev	CT2-5	D1-S27-S80-S41-S111-S107	CT1E-19	D5-S60-S26-S18-S81-S24-S29-S43-S38-S53-S57-S32-S12-S39-S83-S48-S72-S14-S115-S78-S74-S90-S94-S6-S9-S95-S12-S9-S95-S20
4014	2005	Gravis	Asymptomatic	Minsk	CT2-6	D1-S34-S37-S27-S80-S103-S41-S111-S107	CT1E-6	D4-S97-S7-S65-S82-S51-S36-S5-S8-S17-S58-S106-S86-S54-S19-S28-S2-S71-S28-S2-S85-S71-S61-S42-S98-S46-S70-S64-S66-S91
4938	2009	Gravis	Diphtheria	Minsk	CT2-6	D1-S34-S37-S27-S80-S103-S41-S111-S107	CT1E-6	D4-S97-S7-S65-S82-S51-S36-S5-S8-S17-S58-S106-S86-S54-S19-S28-S2-S71-S28-S2-S85-S71-S61-S42-S98-S46-S70-S64-S66-S91
4728	2007	Gravis	Asymptomatic	Minsk	CT2-7	D1-S37-S27-S80-S103-S41-S111-S107	CT1E-6	D4-S97-S7-S65-S82-S51-S36-S5-S8-S17-S58-S106-S86-S54-S19-S28-S2-S71-S28-S2-S85-S71-S61-S42-S98-S46-S70-S64-S66-S91
5073	2010	Gravis	Diphtheria	Minsk	CT2-7	D1-S37-S27-S80-S103-S41-S111-S107	CT1E-18	D5-S60-S26-S18-S53-S57-S32-S39-S83-S48-S72-S14-S12-S115-S78-S74-S90-S94-S6-S9-S95-S12-S9-S95-S20
5052	2010	Gravis	Asymptomatic	Minsk	CT2-7	D1-S37-S27-S80-S103-S41-S111-S107	CT1E-6	D4-S97-S7-S65-S82-S51-S36-S5-S8-S17-S58-S106-S86-S54-S19-S28-S2-S71-S28-S2-S85-S71-S61-S42-S98-S46-S70-S64-S66-S91

Additional File 2: Table S5 continues…

Strain	Year	Biovar	Clinical information	Country	DRA type	DRA locus	DRB type	DRB locus
4800	2008	Gravis	Diphtheria	Minsk	CT2-7	D1-S37-S27-S80-S103-S41-S111-S107	CT1E-20	D5-S95-S53-S57-S32-S39-S83-S48-S72-S14-S115-S78-S20-S74-S90-S94-S26-S18-S81-S24-S29-S43-S38
1072	1998	Gravis	Diphtheria	Minsk	CT2-8	D1-S44-S27-S80-S103-S41-S111-S107	CT1E-13	D4-S97-S7-S65-S82-S8-S17-S58-S106-S54-S19-S86-S42-S98-S46-S70-S64-S66-S91
NCTC 13129	1997	Gravis	Diphtheria	UK	CT2-9	D1-S89-S27-S80-S103-S41-S111-S107	CT1E-5	D4-S97-S65-S82-S8-S17-S58-S106-S54-S19-S28-S2-S86-S85-S71-S28-S2-S85-S71-S61-S42-S98-S46-S70-S91-S91-S7
933a	1997	Gravis	Sore throat	Minsk	CT2-10	D1-S45-S113-S110	CT1E-19	D5-S60-S26-S18-S81-S24-S29-S43-S38-S53-S57-S32-S12-S39-S83-S48-S72-S14-S115-S78-S74-S90-S94-S6-S9-S95-S12-S9-S95-S20
2789	2003	Gravis	Sore throat	Grodno	CT2-11	D3-S33-S3-S21-S63-S92-S55	CT1E-10	D4-S97-S7-S65-S82-S8-S17-S58-S106-S54-S19-S28-S86-S2-S85-S71-S28-S2-S85-S71-S61-S42-S98-S46-S70-S64-S66-S91
4299	2006	Gravis	Diphtheria	Vitebsk	CT2-11	D3-S33-S3-S21-S63-S92-S55	CT1E-16	D5-S60-S24-S29-S43-S38-S53-S57-S32-S39-S83-S48-S12-S72-S14-S115-S78-S74-S90-S94-S6-S9-S95-S20-S26-S18-S81
2225	2001	Gravis	Diphtheria	Brest	CT2-11	D3-S33-S3-S21-S63-S92-S55	CT1E-17	D5-S60-S24-S29-S43-S38-S53-S57-S32-S39-S83-S48-S95-S72-S14-S115-S94-S12-S9-S95-S20-S26-S18-S81
4525	2006	Gravis	Diphtheria	Minsk	CT2-12	D3-S33-S3-S21-S63-S92-S55-S114	CT1E-7	D4-S97-S7-S65-S82-S51-S36-S5-S8-S17-S58-S106-S86-S54-S19-S28-S2-S85-S71-S61-S42-S98-S46-S70-S64-S66-S91
4720	2007	Gravis	Diphtheria	Minsk	CT2-12	D3-S33-S3-S21-S63-S92-S55-S114	CT1E-8	D4-S97-S7-S65-S82-S51-S36-S5-S8-S17-S58-S106-S86-S54-S19-S28-S42-S98-S46-S70-S64-S66-S91
2278a	2001	Gravis	Sore throat	Minsk	CT2-13	D3-S33-S3-S21-S63-S92-S55-S35-S30	CT1E-2	D4-S97-S19-S28-S86-S42-S98-S91-S17-S58-S106-S54
1006	1998	Gravis	Sore throat	Minsk	CT2-13	D3-S33-S3-S21-S63-S92-S55-S35-S30	CT1E-15	D5-S60-S18-S81-S24-S29-S43-S38-S53-S57-S32-S39-S12-S83-S78-S74-S90-S94-S6-S12-S6-S9-S95-S20-S26
700a	1997	Gravis	Diphtheria	Minsk	CT2-14	D3-S33-S3-S21-S63-S92-S55-S68	CT1E-10	D4-S97-S7-S65-S82-S8-S17-S58-S106-S54-S19-S28-S86-S2-S85-S71-S28-S2-S85-S71-S61-S42-S98-S46-S70-S64-S66-S91
2803	2004	Gravis	Asymptomatic	Minsk	CT2-15	D3-S33-S3-S21-S63-S92-S92-S55-S10	CT1E-3	D4-S97-S5-S8-S17-S58-S106-S54-S19-S28-S2-S85-S86-S71-S28-S2-S85-S71-S61-S42-S98-S46-S70-S64-S66-S36
2393	2002	Gravis	Sore throat	Minsk	CT2-16	D3-S33-S3-S92-S55-S52-S50	CT1E-15	D5-S60-S18-S81-S24-S29-S43-S38-S53-S57-S32-S39-S12-S83-S78-S74-S90-S94-S6-S12-S6-S9-S95-S20-S26
1438	1999	Mitis	Asymptomatic	Grodno	-	-	CT1E-19	D5-S60-S26-S18-S81-S24-S29-S43-S38-S53-S57-S32-S12-S39-S83-S48-S72-S14-S115-S78-S74-S90-S94-S6-S9-S95-S12-S9-S95-S20
4502	2006	Gravis	Asymptomatic	Gomel	CT2-17	D2-S116-S25-S77-S31-S16-S93-S93-S16		
209	1996	Gravis	Asymptomatic	Minsk	CT2-18	D2-S40-S67-S49-S15-S16		
2379	2002	Belfanti	Sore throat	Mogilev	CT2-19	D2-S75-S15-S101		
4499	2006	Belfanti	Sore throat	Gomel	CT2-20	D2-S75-S40-S101-S15-S101		
5171	2012	Mitis	Sore throat	Minsk	CT2-21	D2-S75-S40-S16		
1137	1998	Belfanti	Sore throat	Minsk	CT2-22	D2-S75-S40-S16-S15-S101		
1556	1999	Belfanti	Sore throat	Minsk	CT2-22	D2-S75-S40-S16-S15-S101		
1734	2000	Belfanti	Sore throat	Mogilev	CT2-22	D2-S75-S40-S16-S15-S101		
4069	2005	Belfanti	Asymptomatic	Vitebsk	CT2-22	D2-S75-S40-S16-S15-S101		
5162	2012	Gravis	Sore throat	Brest	CT2-23	D2-S75-S76-S67-S79-S49-S15-S16-S16-S22-S22		
5191	2013	Gravis	Sore throat	Minsk	CT2-24	D2-S79-S116-S11-S16		

Additional File 2: Table S5 continues…

Strain	Year	Biovar	Clinical information	Country	DRA type	DRA locus	DRB type	DRB locus
728	1997	Mitis	Sore throat	Minsk	CT2-25	D6-S100-S1-S117-S87-S4		
1054	1998	Gravis	Asymptomatic	Gomel	CT2-26	D6-S117-S102-S59-S88-S87-S4		
768a	1997	Gravis	Sore throat	Brest	CT2-27	D6-S117-S1-S88-S87-S4		
2399	2002	Gravis	Sore throat	Minsk	CT2-28	D6-S117-S84-S104		
4727	2007	Gravis	Sore throat	Minsk	CT2-28	D6-S117-S84-S104		
4994	2009	Gravis	Sore throat	Brest	CT2-28	D6-S117-S84-S104		
2163	2001	Gravis	Asymptomatic	Minsk	CT2-28	D6-S117-S84-S104		
2650	2002	Mitis	Sore throat	Minsk	CT2-29	D6-S117-S87-S4-S104		
2926	2004	Gravis	Asymptomatic	Gomel	CT2-30	D6-S23-S109-S56-S88-S87-S4		
74	1996	Mitis	Asymptomatic	Mogilev	CT2-31	D6-S47-S1-S117-S87-S4		
631	1997	Belfanti	Sore throat	Vitebsk	CT2-31	D6-S47-S1-S117-S87-S4		
5130	2011	Belfanti	Sore throat	Vitebsk	CT2-31	D6-S47-S1-S117-S87-S4		
4766	2008	Belfanti	Sore throat	Gomel	CT2-32	D6-S47-S1-S47-S87-S4		
5201	2014	Mitis	Sore throat	Minsk	CT2-33	D6-S47-S1-S87-S4		
4663	2007	Belfanti	Sore throat	Gomel	CT2-34	D6-S47-S87-S4-S105		
987	1998	Mitis	Sore throat	Gomel	CT2-35	D6-S4-S104		
2370	2001	Mitis	Sore throat	Minsk	CT2-35	D6-S4-S104		
2686	2003	Mitis	Sore throat	Minsk	CT2-35	D6-S4-S104		
2915	2004	Mitis	Sore throat	Vitebsk	CT2-35	D6-S4-S104		
4288	2006	Mitis	Diphtheria	Minsk	CT2-35	D6-S4-S104		
4552	2006	Mitis	Sore throat	Vitebsk	CT2-35	D6-S4-S104		
4726	2007	Mitis	Sore throat	Minsk	CT2-35	D6-S4-S104		
4774	2008	Mitis	Sore throat	Minsk	CT2-35	D6-S4-S104		
5005	2009	Mitis	Sore throat	Gomel	CT2-35	D6-S4-S104		
5085	2010	Mitis	Sore throat	Mogilev	CT2-35	D6-S4-S104		
5138	2011	Gravis	Sore throat	Brest	CT2-36	D6-S69-S4-S73-S73-S117-S117-S1-S96-S56-S88		
ISS 4060	1999	Gravis	Pharyngitis/tonsilitis	Italy	CT2-37	D6-S99-S117-S1-S109-S56-S88-S87-S87-S4		

Note: Direct-repeat and spacer sequences from CRISPR loci were extracted using CRISPRFinder [1]. All direct-repeat sequences and spacer sequences were separately aligned using T-Coffee [2] at the Ressource Parisienne en BioInformatique Structurale server (http://mobyle.rpbs.univ-paris-diderot.fr/). ML trees were calculated from the alignment of direct-repeat sequences with JC substitution model and from the alignment of spacer sequences with HKY model, each with 100,000 SH-like approximate likelihood ratio tests (SH-aLRT) and 100,000 ultrafast bootstrap iterations using IQ-Tree [3]. An allele number was assigned to each group of direct-repeats and spacers in the phylogenetic tree with a phylogenetic distance of zero.

References

1. Grissa I, Vergnaud G, Pourcel C: **CRISPRFinder: a web tool to identify clustered regularly interspaced short palindromic repeats**. *Nucleic Acids Res* 2007, **35**(Web Server issue):W52-57.
2. Notredame C, Higgins DG, Heringa J: **T-Coffee: A novel method for fast and accurate multiple sequence alignment**. *J Mol Biol* 2000, **302**(1):205-217.
3. Nguyen LT, Schmidt HA, von Haeseler A, Minh BQ: **IQ-TREE: a fast and effective stochastic algorithm for estimating maximum-likelihood phylogenies**. *Mol Biol Evol* 2015, **32**(1):268-274.
