# Supplementary material for: Genomic analysis of endemic clones of toxigenic and non-toxigenic Corynebacterium diphtheriae in Belarus during and after the major epidemic in 1990s
Source: BMC Genomics. 2017 Nov 13;18:873. doi: 10.1186/s12864-017-4276-3 (PMC5683216; doi:10.1186/s12864-017-4276-3)
Supplement: Supplementary file 3 — A ML tree from core genomic alignment of ST5 strains; Figure S2. Gubbins analysis of recombination in the core genome of C. diphtheriae. Predicted recombination events on internal branches are shown in red and those occurred at terminal branches are shown in blue; Figure S3. A plot of root-to-tip divergence (Y-axis) against the sampling dates (X-axis); A. within ST5 and B. within ST8; Figure S4. A. CDS BLAST map of ST5 using strain ISS 4060 as the reference. B. CDS BLAST map of ST8 using strain NCTC 13129 as the reference; Figure S5. A ML tree from the binary data of the presence or absence of genes in the accessory genome. The scale bar with a distance of 0.1 represents the difference of 341.7 genes; Figure S6. A plot showing the average global vaccine coverage, reported vaccination in Belarus and the reported number of diphtheria cases between 1992 and 2015. (PDF 5948 kb) [file 12864_2017_4276_MOESM3_ESM.pdf]

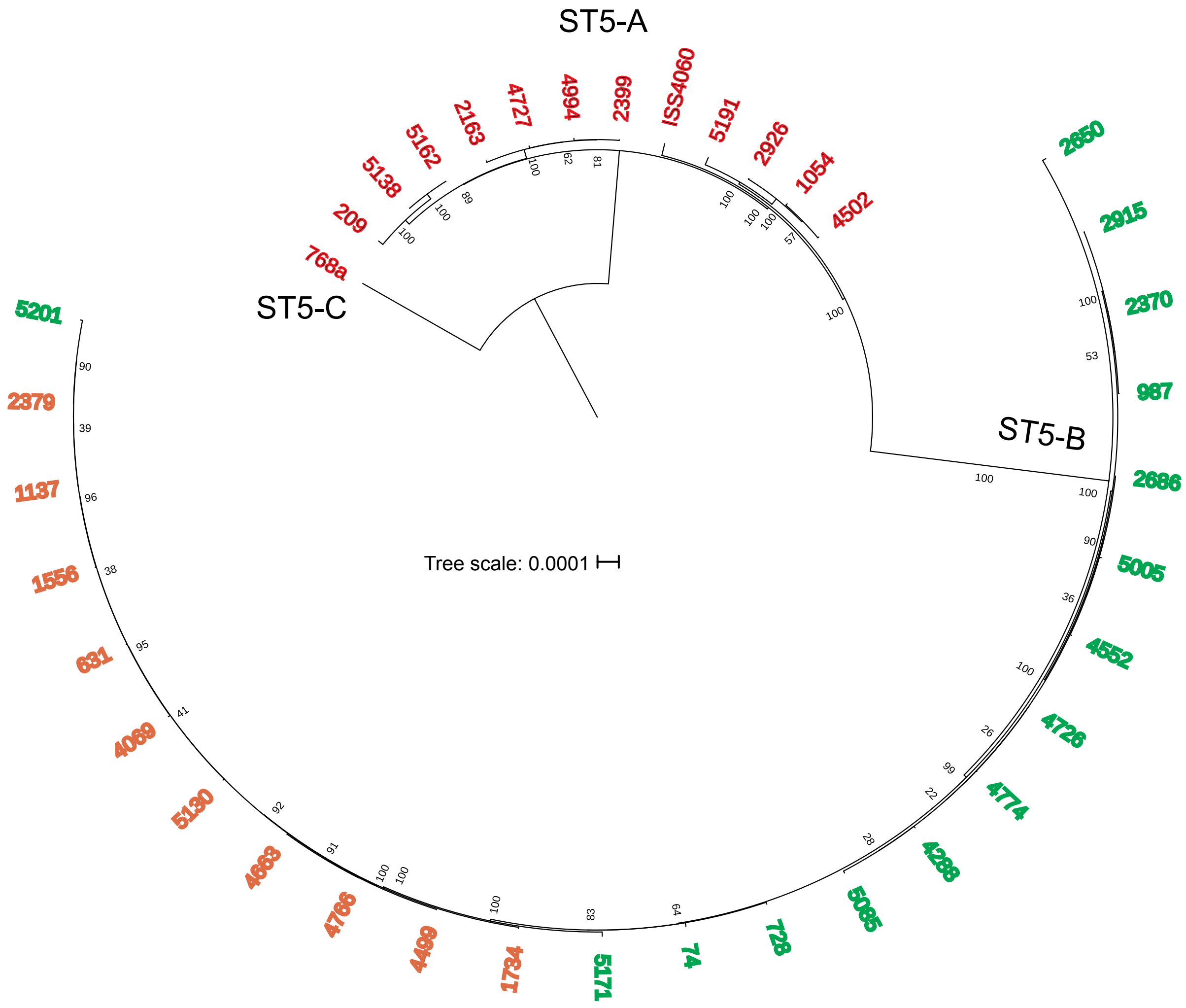

Additional File 3: Fig S1. A ML tree from core genomic alignment of ST5 strains. The strain designations of isolates of biovar belfanti, gravis and mitis are presented in light brown, red, and green colour, respectively.

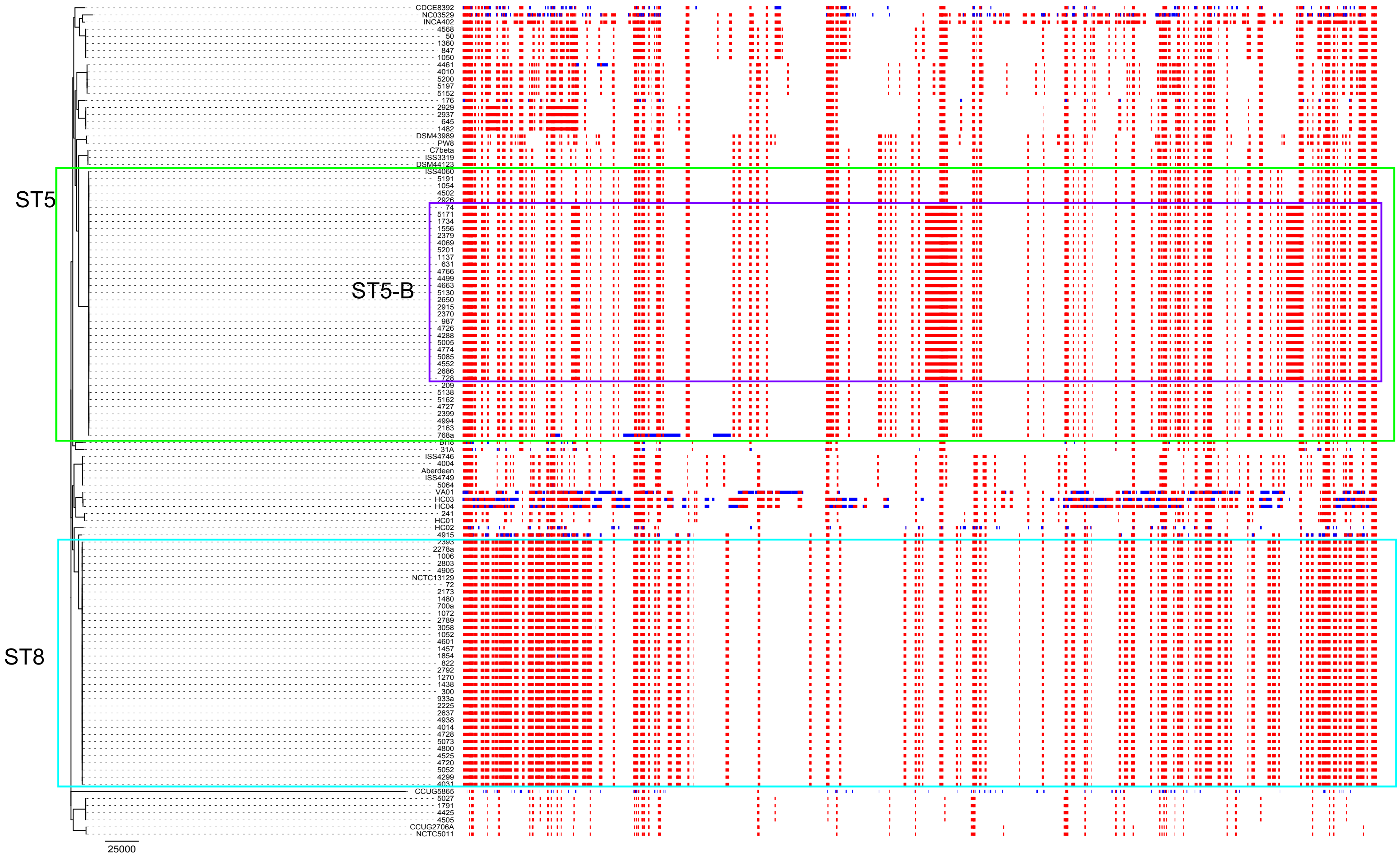

Additional File 3: Fig S2. Gubbins analysis of recombination in the core genome of *C. diphtheriae*. Predicted recombination events on internal branches are shown in red and those occurred at terminal branches are shown in blue. isolates within ST5, subgroup ST5-B and ST8 are highlighted within green, purple and cyan rectangles.

A. ST5

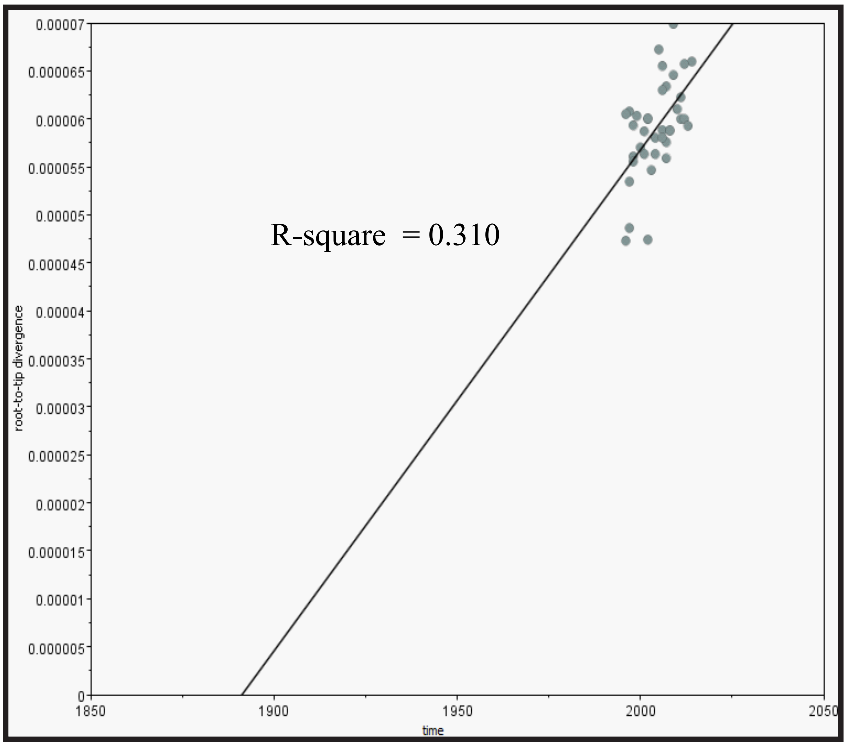

B. ST8

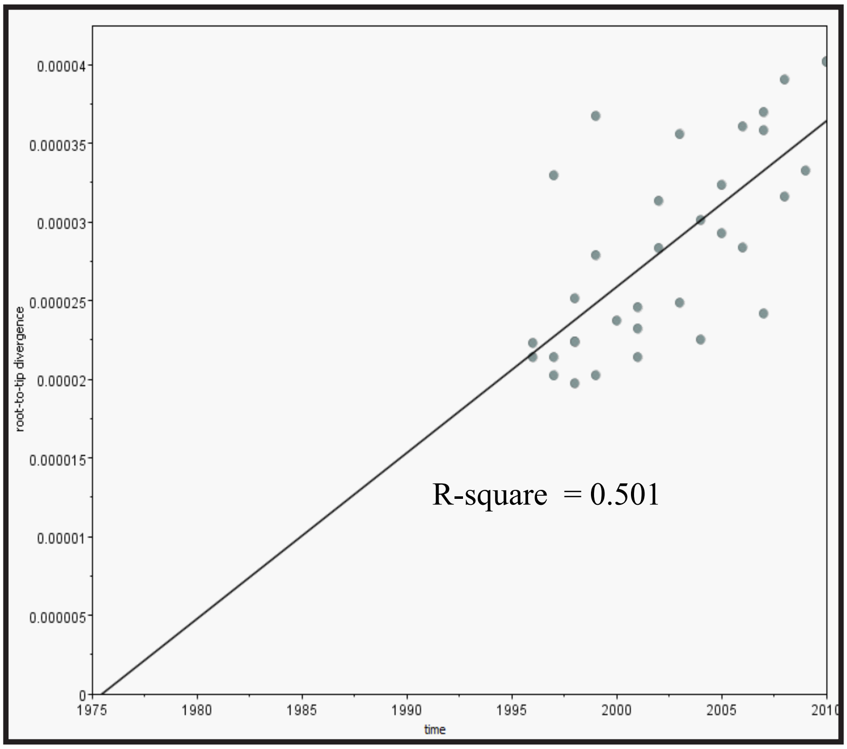

Additional File 3: Fig S3. A plot of root-to-tip divergence (Y-axis) against the sampling dates (X-axis); A. within ST5 and B. within ST8.

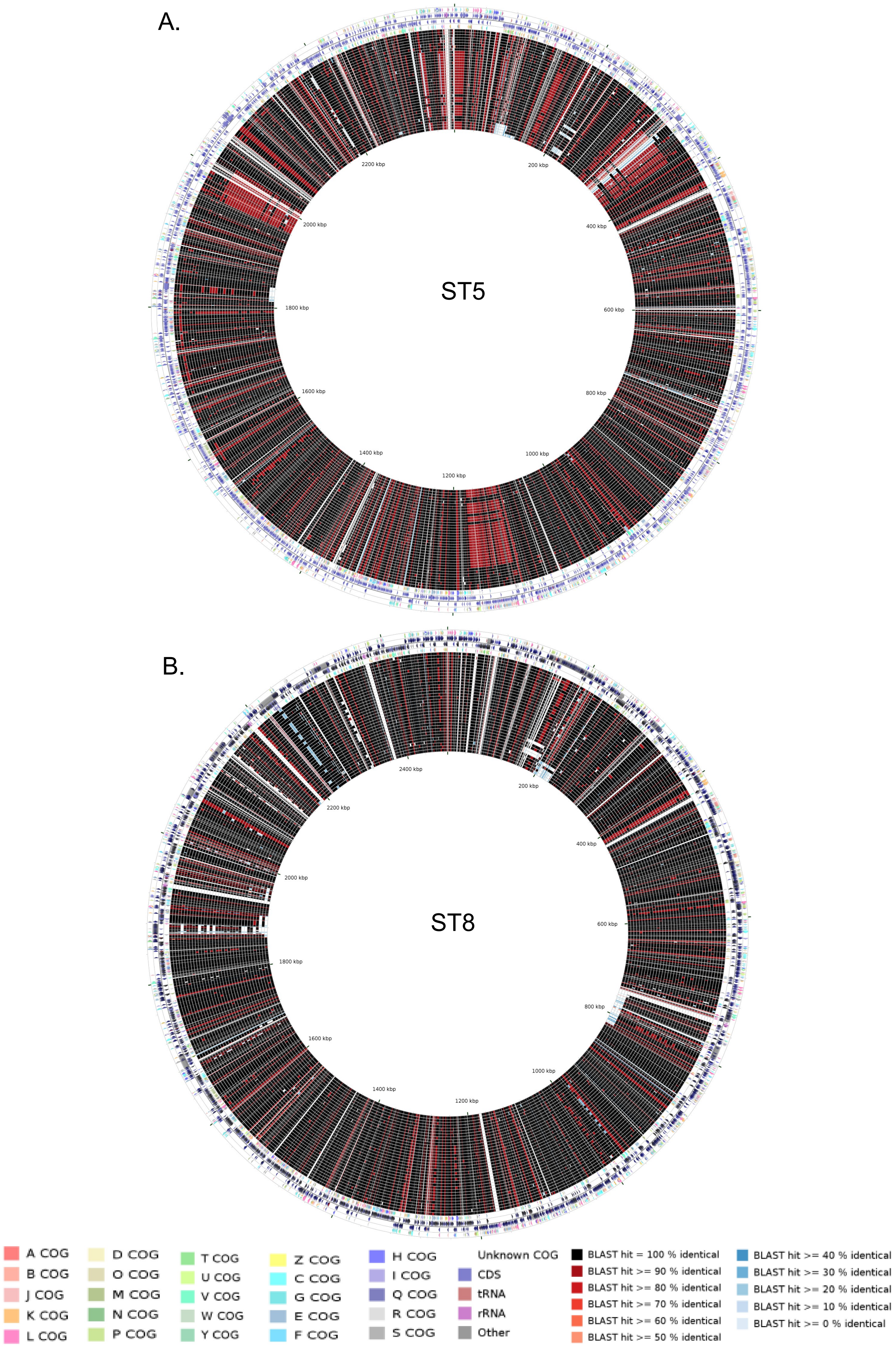

Additional File 3: Fig S4. A. CDS BLAST map of ST5 using strain ISS 4060 as the reference. B. CDS BLAST map of ST8 using strain NCTC 13129 as the reference.

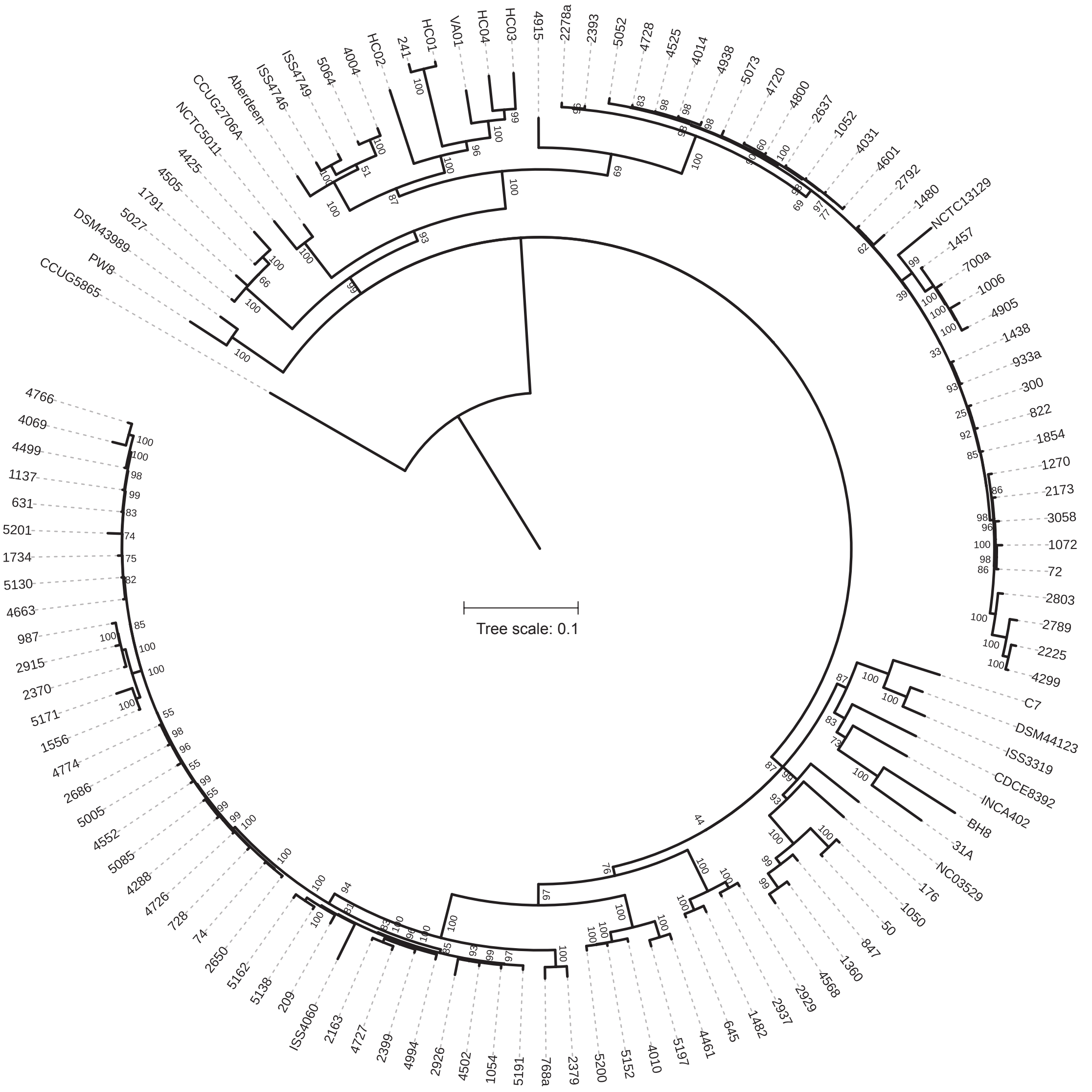

Additional File 3: Fig S5. A ML tree from the binary data of the presence or absence of genes in the accessory genome using GTR2+FO+ASC+R5 model according to Bayesian Information Criterion with 1,000 ultrafast bootstrap iterations and 1,000 SH-like approximate likelihood ratio test iterations. The scalebar with a distance of 0.1 represents the difference of 341.7 genes.

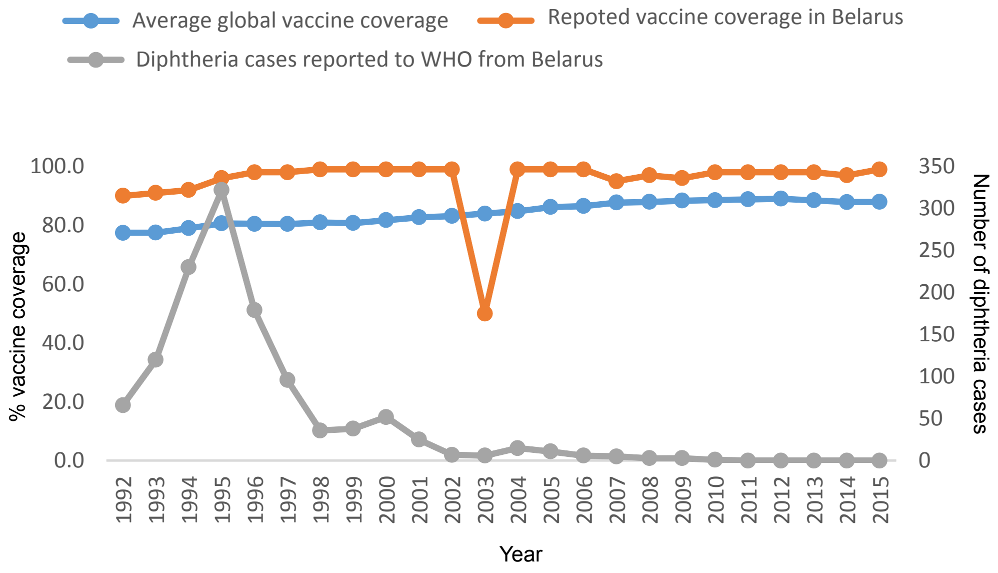

Additional File 3: Fig S6. A plot showing the average global vaccine coverage, reported vaccination in Belarus and the reported number of diphtheria cases between 1992 - 2015 (WHO, 2017; http://www.who.int/topics/diphtheria/en/ and http://www.who.int/immunization/monitoring_surveillance/data/en/; accessed February 2017)
